# Recurrent pulmonary infection leads to the diagnosis of triple A syndrome: a case report

**DOI:** 10.1186/s13256-022-03509-1

**Published:** 2022-07-28

**Authors:** Sawssan Ali, M. Subhi Murad, Humam Hamdan, Wael Nakawa

**Affiliations:** 1 Pediatric Pulmonology, Damascus University Children’s Hospital, Damascus, Syria; 2 Faculty of Medicine, Damascus University, Damascus, Syria

**Keywords:** Alacrima, Achalasia, Adrenal insufficiency, Triple A syndrome, Case report

## Abstract

**Introduction:**

Triple A syndrome is a very uncommon disease marked by a triad of adrenocorticotrophic hormone (ACTH)—resistant features: adrenal insufficiency, alacrimia, and achalasia. It presents in several clinical forms with undetermined incidence and shows an autosomal pattern of inheritance. It is caused by a variety of mutations in the AAAS genes which encode a protein of unknown function called ALADIN. Diagnosis depends on clinical manifestations, laboratory test results, imaging and endoscopic findings, and Schirmer’s test. The treatment includes artificial tears, glucocorticoid replacement therapy, and treatment of achalasia.

**Case presentation:**

A 12-year-old Syrian girl was referred to Damascus University’s Children’s Hospital for recurrent pulmonary infection. Her mother had noted an absence of tears when crying since birth, diffused pigmentations since birth, especially on the cheeks and genitals, recurrent vomiting of both solid and liquid foods, and recurrent exacerbations of bronchitis and recurrent pneumonia. ACTH and blood cortisol levels indicated an adrenal insufficiency, chest computed tomography and barium swallow test results indicated achalasia, tear break-up time as well as eye examination indicated alacrimia, which led to the diagnosis of triple A syndrome. Treatment included Heller cardiomyotomy, artificial tears, and hydrocortisone (15–30 mg/m^2^), as well as continuous observation of ACTH levels.

**Discussion:**

Triple A syndrome (which is characterized by the triad of achalasia, alacrima, adrenal insufficiency) is a rare multisystem disease. It has a genetic background and is potentially fatal. This syndrome is often misdiagnosed, especially in regions where it is expected to have a high prevalence rate (regions with documented cases and high rate of consanguinous marriage), This study is the first documentation of triple A syndrome in Syria, a country where consanguineous marriage is common. This syndrome should be kept in mind when a child presents with one or more of its characteristic features.

## Introduction:

Triple A syndrome (Allgrove syndrome or 3A syndrome) is a rare medical condition, and the classic form of this multisystem disease is defined by the presence of a triad of very specific features: adrenocorticotrophic hormone (ACTH) resistance (adrenal insufficiency), alacrimia, and achalasia [1]. Adrenal insufficiency refers to a condition in which the receptors of the ACTH hormone are no longer sensitive to ACTH hormone, which leads to glucocorticoid deficiency [2]. Achalasia is an esophageal motility impairment that results in the lower esophageal sphincter being unable to relax and let food enter the stomach, which can cause dysphagia [3]. Alacrimia describes the condition of decreased amount of tear production [1]. Other forms of this syndrome have been described, with the term 2A syndrome describing a condition with two out of the three classic manifestations. If the triad is associated with autonomic neuropathy, it is then called 4A syndrome. When there are amyotrophy and other neurological manifestations it is called 5A syndrome [1].

The actual incidence rate of triple A syndrome is difficult to determine due to its rarity. It has a recessive autosomal pattern of inheritance. Parental consanguinity is a risk factor for this syndrome [1].

The affected gene in this syndrome is the AAAS gene which has a penetrance of close to 100%. Mutations of the AAAS gene, which is localized on chromosome 12q13, play a key role in the etiology, and many genetic mutations have been detected. The AAAS gene consists of 16 exons and encodes a protein called ALADIN (alacrimia-achalasia-adrenal insufficiency neurologic), which belongs to the WD-repeat family. The exact function of this protein is still unknown, although it is a part of the nuclear pore complex [1].

Pathologically, the lacrimal glands show loss of serous-secreting cells, atrophy in the adrenal glands, and muscular hypertrophy. Loss of ganglion cells have been seen in the distal esophagus [1].

The age of onset of the clinical manifestations vary, with alacrimia present at birth and achalasia and adrenal insufficiency appearing late in the first decade of life. There is no specific genotype-phenotype correlation in the syndrome, and patients with the same mutation show different manifestations. Symptoms and signs may include dehydration-induced keratopathy, recurrent or chronic pulmonary disease, hypoglycemia, mental retardation, microcephaly, palmar and plantar hyperkeratosis, hyperpigmentations, and other neurological and structural abnormalities [1].

Alacrimia is diagnosed by Schirmer’s test. The diagnosis of adrenal insufficiency can be established by a 250-µg ACTH stimulation test. Esophageal manometry, which is typically performed along with Barium meal examination and gastroesophagoscopy, are used to diagnose achalasia [1]. Differential diagnosis includes adrenoleukodystrophy (ALD), familial glucocorticoid deficiency (FGD), and Sjogren syndrome [1].

The treatment of triple A syndrome requires the treatment of each of the triad. Alacrimia is treated with artificial tears. Hydrocortisone is the drug of choice for the treatment of adrenal insufficiency in glucocorticoid replacement therapy. Achalasia can be treated by surgical (Heller’s esophago cardiomyotomy) and nonsurgical (pneumatic dilatation) treatments [1].

Management of the allgrove syndrome is usually multidisciplinary, involving multiple specialties and a high level of co-ordination among the primary care provider, ophthalmologist, endocrinologist, gastroenterologist, and possibly a general surgeon [4].

This case report adds to the limited body of scientific literature currently available on this rare syndrome, and is the first documented case of triple A syndrome in Syria, where we suspect that this syndrome has a higher incidence than it’s global incidence due to the widespread practice of onsanguineous marriage.

## Case presentation

A 12-year-old Syrian girl was referred to the Damascus University’s Children’s Hospital for recurrent pneumonia and bronchitis. Her mother had noted an absence of tears when crying since birth, diffused pigmentations since birth, especially on the cheeks and genitals, repeated vomiting of both solid and liquid foods, and asthma. The mother also mentioned that the child’s cousin had had similar symptoms.

On physical examination, the child showed fatigue with pigmentation on her lips, hyperpigmentation on the nose and under the right eye, signs of growth failure (weight: 25.5 kg; height: 133 cm), dyspnea with a respiratory rate of 40–45 breaths per minute, substernal intercostal retractions, hyperpigmentation on genitals, digital clubbing, and hyperkeratosis on the palms and soles. Results of the neurological examination were normal, and the patient’s psychomotor development was also normal.

The blood tests results were: Na^+^, 136 mmol/l; creatinine, 0.6 mg/dl; and urea, 21 mg/dl. Immunoglobulin levels were normal.

**Figure:**
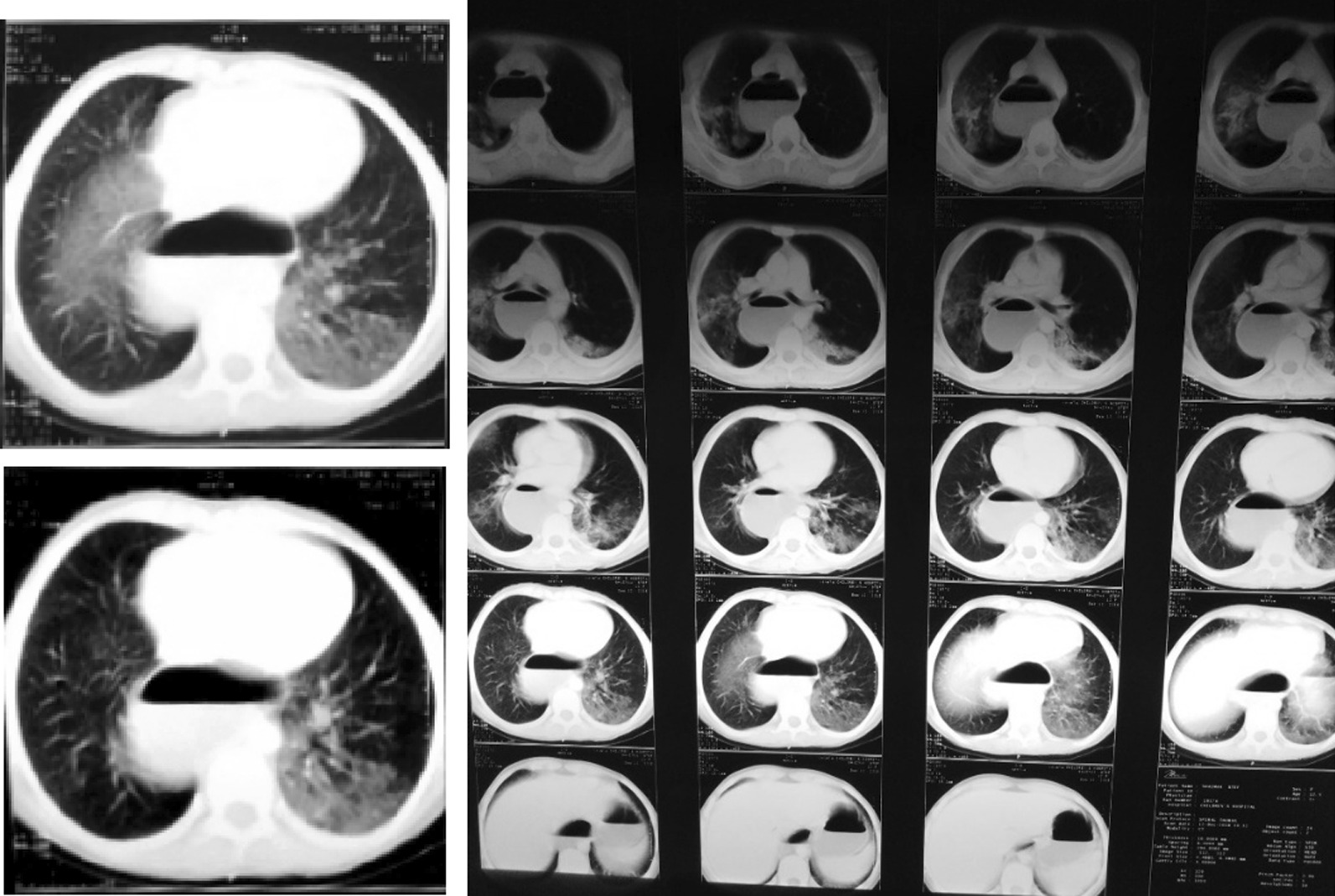

Computed tomography (CT) scan of the thorax showed massive dilation of the esophagus and bilateral pulmonary infiltration.

**Figure:**
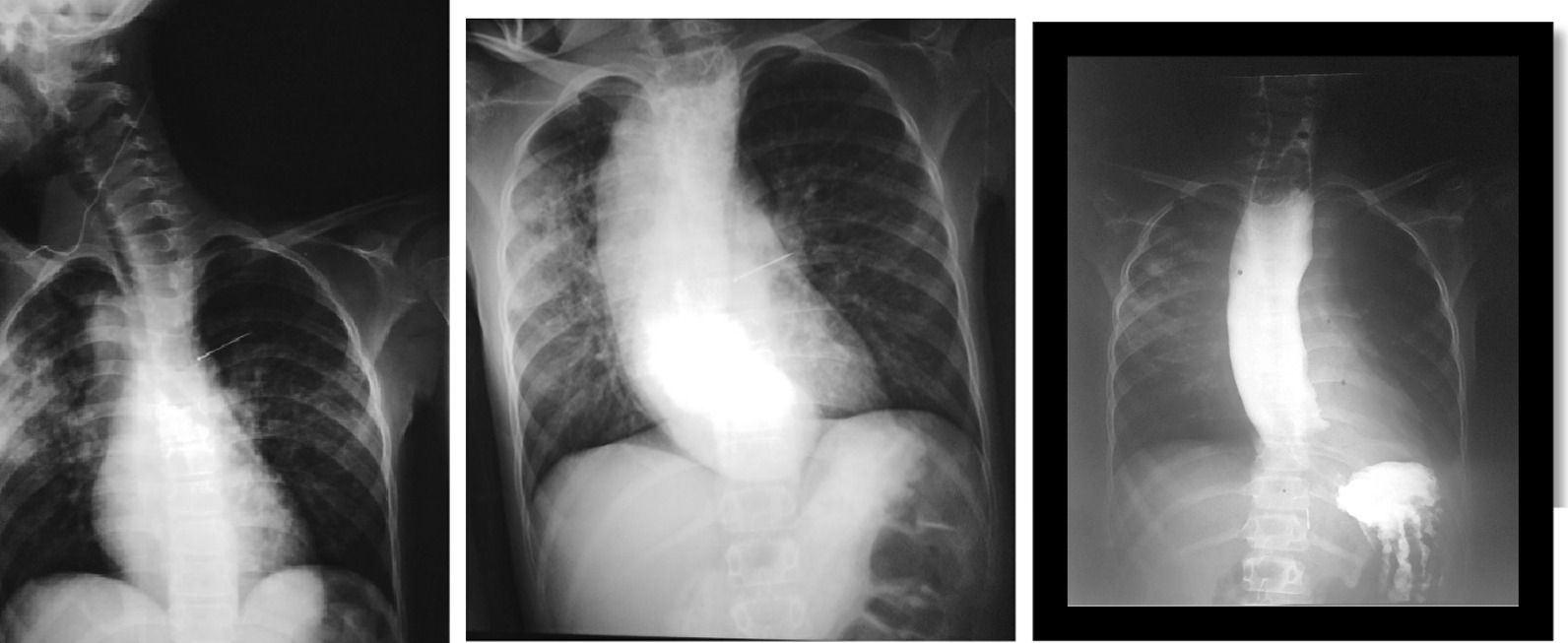

Barium swallow test showed massive dilation in the esophagus associated with a tightening in the lower esophageal sphincter (bird beak sign).

Esophagogastroduodenoscopy showed severe dilation in the esophageal lumen with a contraction in the lower esophageal sphincter that continues to the stomach, which confirmed the diagnosis. The patient’s cortisol and ACTH levels were tested to determine the cause of the cutaneous lesions and the diagnosis of adrenal insufficiency (Table 1).

**Table 1:** Patient’s cortisol and adrenocorticotrophic hormone levels

Cortisol and ACTH	Test results	Normal range
ACTH	49.76*10^−6^ microgram/ml	4.3–22.7 × 10^−6^ μg/ml
Cortisol	13.09 mcg/dl	2–14 mcg/dl (p.m)

*ACTH* Adrenocorticotrophic hormone,* mcg* microgram,* dl* decilitre,* ml* milliliter,* μg* Microgram,* p.m* post meridiem

An opthalmologic consultation was done to determine the cause of tear absence. The patient was found to have visual ability of 10/10 in both eyes, eye dryness was present, and tear break-up time was 2 seconds. Defects in the lower epithelium of the eyes were also present. Schirmer’s test was unavailable. Based on these findings, alacrimia was diagnosed.

The patient was diagnosed with triple A syndrome, which consists of alacrimia, achalasia, and adrenal insufficiency and treated accordingly. For the alacrimia, artificial tears or Viscotears liquid gel drops 5 times a day (Bausch & Lomb, Laval, QB, Canada) were prescribed. For the adrenal insufficiency, hydrocortisone (15–30 mg/m^2^) was prescribed, and ACTH levels were checked every 6 months. For the achalasia, Heller cardiomyotomy was performed, with a 7-cm longitudinal myotomy.

The medical team contacted the patient after 3 years. Her mother reported the disappearance of the skin lesions, and that the vomiting has stopped (Heller’s operation outcome) and that she is maintaining the child on her medication.

## Discussion

Triple A syndrome is a rare, recessive autosomal, multisystem disease with variable phenotype. It has an estimated prevalence rate of 1 in 1 million individuals, but its true prevalence rate is hypothesized to be higher due to missed diagnosis [4].

It involves mutations in the AAAS gene which is located on chromosome 12q13 [5], which lead to impaired function of the ALADIN protein [1]. Although it has variable phenotypic manifestations, triple A syndrome is most likely to manifest in pediatric patients as the triple A triad, which includes achalasia, alacrimia, and adrenal insufficiency. However, other manifestations, such as neurological manifestations, could appear later at an older age [1], which explains the lack of neurological manifestations in our patient.

Triple A syndrome can be life-threatening when misdiagnosed; as well, an early diagnosis can widely affect the prognosis [5]. Its diagnosis depends on both physical examination findings and adrenal tests [1].

Although 15% of triple A syndrome cases show mineralocorticoid deficiency [6], normal sodium and potassium levels make up for the lack of aldosterone testing.

According to Flokas* et al*. [4], the achalasia present in triple A syndrome manifests in dysphagia, failure to thrive, regurgitation, and pulmonary symptoms (cough, hoarseness, wheezing, and recurrent pneumonia). The treatment of choice can be surgical, through Heller cardiomyotomy, or non-surgical, using pneumatic esophageal dilation. A close follow-up is highly recommended. Alacrimia is the least symptomatic of the triad, but the most prevalent finding in patients with triple A syndrome. Alacrimia is treated with artificial tears and lubricants. Adrenal insufficiency is the most fatal finding in this syndrome due to acute hypoglycemia. It often manifests in hyperpigmentation of the skin and mucosae and hypoglycemic shock. Treatment requires a close follow-up by a pediatric endocrinologist and administration of short-acting glucocorticoid. Other manifestations, such as palmoplantar hyperkertosis, have been reported in some cases [1]. Only a few dozen published reports of this syndrome can be found in the medical literature, mostly as case reports and case reviews with some notable exceptions.

Allgrove* et al*. was the first to describe this syndrome in 1978. These authors noticed the main characteristics of this disease in four children (2 pairs of siblings from separate families), and this syndrome was then named after them (Allgrove syndrome) [7]. In 1996 Weber and colleagues identified the genetic mutation that causes this syndrome using genome linkage scan [5]. Flokas* et al*. published a significant review paper in 2019 that covered most published studies on this syndrome, and recommended treatment and follow-up, noting the importance and potential lethality of misdiagnosed cases [4].

Two of the biggest strengths of our case report is that it adds to the limited scientific literature on this rare syndrome, as well as being the first documented case of triple A syndrome in Syria. However, we do suspect that this syndrome does have a higher incidence in Syria than the globally expected incidence due due to widespread consanguineous marriage in this country.

## Conclusion

Although triple A (Allgrove) syndrome is a rare disease, this study demonstrates its presence in Syrian ethnicity, which has a high rate of consanguineous marriage. The possibility of this syndrome should be kept in mind when a child presents with achalasia or alacrimia in order to prevent potential mortality due to misdiagnosis.

## Data Availability

Not applicable.
